# The bovine lactation genome: insights into the evolution of mammalian milk

**DOI:** 10.1186/gb-2009-10-4-r43

**Published:** 2009-04-24

**Authors:** Danielle G Lemay, David J Lynn, William F Martin, Margaret C Neville, Theresa M Casey, Gonzalo Rincon, Evgenia V Kriventseva, Wesley C Barris, Angie S Hinrichs, Adrian J Molenaar, Katherine S Pollard, Nauman J Maqbool, Kuljeet Singh, Regan Murney, Evgeny M Zdobnov, Ross L Tellam, Juan F Medrano, J Bruce German, Monique Rijnkels

**Affiliations:** 1 Department of Food Science and Technology, University of California Davis, One Shields Avenue, Davis, CA 95616, USA; 2 Department of Molecular Biology and Biochemistry, Simon Fraser University, University Drive, Burnaby, BC, V5A 1S6, Canada; 3 Department of Physiology and Biophysics, University of Colorado Denver, Anschutz Medical Center, E. 19th Ave, Aurora CO 80045, USA; 4 Department of Animal Science, Michigan State University, East Lansing, MI 48824-1225, USA; 5 Department of Animal Science, University of California Davis, One Shields Avenue, Davis, CA 95616, USA; 6 Department of Structural Biology and Bioinformatics, University of Geneva Medical School, rue Michel-Servet, 1211 Geneva, Switzerland; 7 CSIRO Livestock Industries, Queensland Bioscience Precinct, Carmody Road, St Lucia, Queensland 4067, Australia; 8 Center for Biomolecular Science and Engineering, University of California Santa Cruz, High St, Santa Cruz, CA 95064, USA; 9 Dairy Science and Technology, AgResearch, Ruakura Research Centre, East Street, Hamilton, 3240, New Zealand; 10 Division of Biostatistics and Gladstone Institutes, University of California San Francisco, Owens St, San Francisco, CA 94158, USA; 11 Bioinformatics, Mathematics and Statistics, AgResearch, Invermay Agricultural Centre, Puddle Alley, Mosgiel 9053, New Zealand; 12 Department of Genetic Medicine and Development, University of Geneva Medical School, rue Michel-Servet, 1211 Geneva, Switzerland; 13 Swiss Institute of Bioinformatics, rue Michel-Servet, 1211 Geneva, Switzerland; 14 Imperial College London, South Kensington Campus, London, SW7 2AZ, UK; 15 Nestlé Research Centre, Vers-chez-les-Blanc CH-1000, Lausanne 26, Switzerland; 16 Department of Pediatrics, Children's Nutrition Research Center, Baylor College of Medicine, Bates Street, Houston TX 77030, USA

## Abstract

**Background:**

The newly assembled *Bos taurus *genome sequence enables the linkage of bovine milk and lactation data with other mammalian genomes.

**Results:**

Using publicly available milk proteome data and mammary expressed sequence tags, 197 milk protein genes and over 6,000 mammary genes were identified in the bovine genome. Intersection of these genes with 238 milk production quantitative trait loci curated from the literature decreased the search space for milk trait effectors by more than an order of magnitude. Genome location analysis revealed a tendency for milk protein genes to be clustered with other mammary genes. Using the genomes of a monotreme (platypus), a marsupial (opossum), and five placental mammals (bovine, human, dog, mice, rat), gene loss and duplication, phylogeny, sequence conservation, and evolution were examined. Compared with other genes in the bovine genome, milk and mammary genes are: more likely to be present in all mammals; more likely to be duplicated in therians; more highly conserved across Mammalia; and evolving more slowly along the bovine lineage. The most divergent proteins in milk were associated with nutritional and immunological components of milk, whereas highly conserved proteins were associated with secretory processes.

**Conclusions:**

Although both copy number and sequence variation contribute to the diversity of milk protein composition across species, our results suggest that this diversity is primarily due to other mechanisms. Our findings support the essentiality of milk to the survival of mammalian neonates and the establishment of milk secretory mechanisms more than 160 million years ago.

## Abstract 2

Comparison of milk protein and mammary genes in the bovine genome with those from other mammals gives insights into the evolution of lactation.

## Background

**Figure 1:**
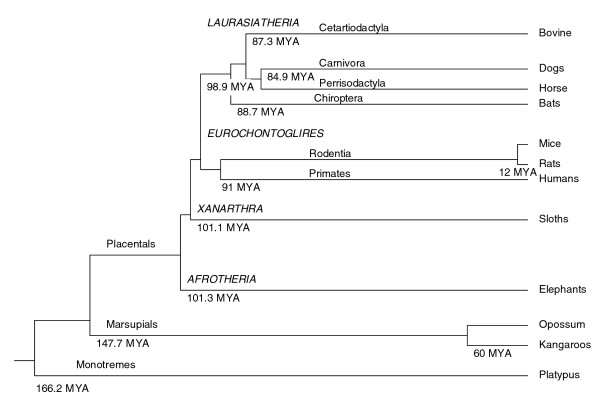
Simplified phylogenetic tree illustrates relationships of representative extant Mammalian species. Estimates in millions of years ago (MYA) of origin of each major branch were derived from Bininda-Emonds *et al*. [2]. The two earliest splits established monotremes, (166.2 MYA), and marsupials and placentals (147.7 MYA). Approximately 50 million years pass before the origination of any extant groups, and then the four placental superorders (italicized capitals) arose within 2.4 million years of each other.

With the arrival of the *Bos taurus *genome assembly, bovine milk and lactation data can be linked to other mammalian genomes for the first time, allowing us to gain additional insight into the molecular evolution of milk and lactation. Mammals are warm-blooded vertebrate animals that nourish their young with milk produced by mammary glands. They first appeared approximately 166 million years ago, but their evolution can be traced back 310 million years when synapsids first branched from amniotes [1]. Two subclasses of mammals evolved, the prototherians and therians. Prototheria are monotremes, mammals that lay eggs; extant species include the platypus and enchidnas. Theria are mammals that bear live young; they are divided into the infraclasses Metatheria or marsupials - which include kangaroos and opossums - and the more common Eutheria or placental mammals - which include, for example, humans, dogs, mice, rats, and bovine species. Figure 1 shows the mammalian phylogenetic tree with approximate divergence times [2,3]. Of the mammalian species listed, high coverage genomic data are available for the platypus (*Ornithorhynchus anatinus*), a prototherian, the opossum (*Monodelphis domestica*), a metatherian, and a number of placental mammals, including human (*Homo sapiens*), rat (*Rattus norvegicus*), mouse (*Mus musculus*), dog (*Canis familiaris*), and now bovine (*Bos taurus*).

The reproductive strategy, developmental requirements of the young, and environment of the maternal-infant pair are thought to drive variation in milk composition among species. Platypus and opossum neonates are embryonic in appearance and dependent on milk for growth and immunological protection during the equivalent of the fetal period in placental mammals [4,5]. In contrast, placental mammals have relatively longer gestation and shorter lactation periods. These reproductive strategies directly impact milk composition as the immature monotreme and marsupial young have different needs with regard to growth, development, and adaptive immunity. Other aspects of the reproductive strategy, such as the length of the lactation period and the maternal nutritional strategy, can also impact milk composition. For example, mammals that fast or feed little during lactation produce milks low in sugar but high in fat to minimize energy and water demands while sustaining nutrient transfer to the young [6]. The data in Table 1 illustrate that even the gross macronutrient composition of milk can be highly variable among species.

**Table 1:** Gross macronutrient composition of mammalian milk

Species	Fat %	Crude protein %	Lactose and sugar %
Bovine [76]	3.7 (3.5-5.5)	3.4	4.6
Dog [77]	9.5	7.5	3.8
Human [76]	4	1	7
Mouse [77]	27	12.5	2.6
Rat [77]	8.8	8.1	3.8
Opossum [78]	7.4	10	10 (oligosaccharides)
Platypus [77]	22.2	8.2	3.7 (difucosylactose)

Because bovine milk is a major human food and agro-economical product, comparison of bovine milk with the milk of other species in the context of the bovine genome sequence is important not only to improve our understanding of mammary evolution but also of bovine milk production and human nutrition. The importance of bovine milk consumption to humans is underscored by the domestication of cattle and the convergent evolution of lactase persistency in diverse human populations [7]. The availability of the bovine genome sequence provides unique opportunities to investigate milk and lactation. Lactation has been studied more extensively in *Bos taurus *than in other species, resulting in extensive milk proteome data, milk production quantitative trait loci (QTL), and over 100,000 mammary-related bovine expressed sequence tags (ESTs).

In the present study, we identified the bovine lactation genome *in silico *and examined its content and organization. Utilizing the genomes of the seven mammals listed above and in Table 1, we investigated gene loss and duplication, phylogeny, sequence conservation, and evolution of milk and mammary genes. Given the conspicuous absence of some known abundant proteins, such as beta-lactoglobulin and whey acidic protein, in the milk of some species [8], we hypothesized that variation in milk composition resides in part in variation in the milk protein genome. We show that gene duplication and genomic rearrangement contribute to changes in the milk protein gene complement of *Bos taurus *and other species. Although the casein proteins are highly divergent across mammalian milks [9,10], we report that milk and mammary genes are more highly conserved, on average, than other genes in the bovine genome. Our findings illustrate the importance of lactation for the survival of mammalian species and suggest that we must look more deeply, perhaps into the non-coding regions of the genome that regulate milk protein gene expression, to understand the species-specificity of milk composition. Among mammals, we find milk proteins that are most divergent have nutritional and immunological functions, whereas the least divergent milk protein genes have functions that are important for the formation and secretion of mammalian milk. High conservation of milk fat globule membrane protein genes among the mammalian genomes suggests that the secretory process for milk production was firmly established more than 160 million years ago.

## Results and discussion

### Milk and mammary gene sets

**Figure 2:**
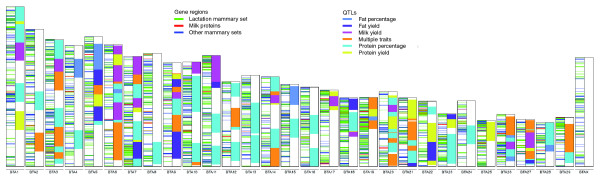
Distribution of milk and mammary genes across all bovine chromosomes. In this chromosome map, each of the 30 bovine chromosomes is illustrated by a pair of columns, with genomic locations of milk and mammary genes in the first column, and milk-trait QTL in the second column. Note that the milk and mammary genes are distributed across all chromosomes.

Two proteome studies of bovine milk [11,12] were used to derive a milk protein gene set of 197 unique genes (see 'Collection of the milk protein set' in Materials and methods). Using 94,136 bovine mammary ESTs, mammary gene sets were created to represent the following developmental stages or conditions: virgin, 3,889 genes; pregnancy, 1,383 genes; lactation, 3,111 genes; involution, 867 genes; and mastitis, 840 genes (see 'Collection of the mammary gene sets' in Materials and methods). In total, 6,469 genes are constituents of one or more of these mammary gene sets, suggesting that one-quarter of all predicted genes are expressed in the mammary gland at some point during the lactation cycle. Genes from the milk protein and mammary gene sets are present on all 29 bovine autosomes and on the X chromosome (Figure 2).

The milk protein gene set is the most extensive curation to date of genes that give rise to milk proteins, the functions of which have not yet been comprehensively studied. To gain insight into the possible molecular functions of milk proteins, the milk protein gene set was analyzed for enriched molecular function Gene Ontology (GO) terms (see Materials and methods). Four significant, minimally redundant molecular function GO terms were identified: 'GTPase activity,' 'GTP binding,' 'pattern recognition receptor activity,' and 'calcium ion binding.' More than 30 milk proteins that were previously isolated in the milk fat globule membrane [11,12] were associated with 'GTPase activity' or 'GTP binding'. GTPases are known to be involved in numerous secretory processes, and for this reason, it seems likely that these proteins have a role in assembly and secretion of the milk fat globule and possibly other milk components. The 'pattern recognition receptor activity' GO term was enriched due to the presence in milk of the cell surface and immune recognition components CD14 [GenBank:NM_174008], TLR2 [GenBank:NM_174197], TLR4 [GenBank:NM_174198], and DMBT1 [GenBank:S78981]. These proteins are involved in the activation of the innate immune system when they associate with cells. Further, the soluble forms of CD14 and TLR2, which can act as decoy receptors for microbial pathogens, could potentially modulate local inflammation following bacterial colonization in the neonate gut [13,14]. Enrichment of the GO term 'calcium ion binding' was expected as many milk proteins are known to bind calcium, a mineral required in abundance by the growing neonate.

Milk is traditionally thought of as a food that provides the neonate with nutrients and some immune protection, such as that provided by immunoglobulins. Prior research also suggests that various milk proteins are resistant to digestion by gastric proteases at physiological pH [15] and that intact or partially intact milk proteins may either express their functions in the neonatal intestinal tract or may be absorbed and act on other organs [16]. To understand what signaling might be possible if milk proteins remain partially or wholly undigested, the milk protein gene set was interrogated for enriched pathway annotations (see 'Pathway analysis' in Materials and methods). The milk protein gene set contains elements of two marginally significant pathways that lead to activation of PPARalpha and LXR, two nuclear receptors involved in sensing nutrients and modifying metabolic responses at the level of gene transcription. Milk proteins that are associated with the LXR/RXR activation pathway include the cell surface or secreted molecules CD14 [GenBank: NM_174008], CD36 [GenBank:NM_174010], TLR4 [GenBank:NM_174198], and MSR1 [GenBank:NM_001113240], the apolipoproteins APOA1 [GenBank:NM_174242] and APOE [GenBank:NM_173991] and the lipid synthesis enzymes ACACA [GenBank:NM_174224] and FASN [GenBank:NM_001012669]. Those associated with the PPARalpha/RXRalpha activation pathway include the cell surface molecule CD36 [GenBank:NM_174010], the endoplasmic reticulum protein disulphide isomerase PDIA3 [GenBank:NM_174333], the apolipoprotein APOA1 [GenBank:NM_174242], the transcription factor STAT5B [GenBank:NM_174617], the heat shock protein HSP90AA1 [GenBank:NM_001012670], the regulator of adenylate cyclase GNAS [GenBank:NM_181021], and two enzymes involved in lipid synthesis, GPD2 [GenBank:NM_001100296] and FASN [GenBank:NM_001012669]. It is likely that the products of these genes, which are well known to be active at metabolic control points in many organs, are active in the mammary gland and then enter the milk via cytoplasmic crescents in the milk fat globules. Keenan and Patton [17] noted that cytoplasmic sampling, as part of milk fat globule formation, is present in all species examined to date, including humans, and that such evolutionary persistence suggests possible benefits for mother or offspring. Further research will be needed to determine whether these proteins are present in milk at sufficient quantities to have a physiological effect in the neonate.

All mammary gene sets were interrogated for enrichment of GO terms or pathway annotations, but the results did not further our knowledge of mammary biology. Consistent with our previous study [18], current GO term annotations were incomplete or generally out of context when applied to the mammary gland. Although bovine EST data indicate that more than 3,000 genes are expressed in the lactating mammary gland, a mere 22 genes are currently annotated with the GO term 'lactation.'

### Bovine milk production QTL

Milk trait QTL delineate gene regions that harbor genes or *cis*-acting elements that are responsible for the milk trait phenotype. The dairy industry has invested enormous resources into the identification of these QTL for milk production traits in bovine, particularly milk yield, protein yield, fat yields, protein percentage, and fat percentage. Reviewing the literature, 238 milk trait QTL were identified for these five traits in 59 references (Additional data files 8-9). Of the 238 QTL, 63 were reported with flanking markers having a median interval size of approximately 17 million base pairs. Following a previously established method [19], the 175 remaining QTL that were reported with only a single peak marker were assigned this median interval size. Some QTL were reported for more than one milk trait; thus, these QTL span only 168 unique genome locations. These milk trait QTL span all 29 autosomes (Figure 2), with the highest densities of QTL occurring on chromosomes 27, 6, 20, and 14 (Additional data file 10). Possible differences in genetic architecture are most obvious between fat and protein percentage traits, where fat percentage QTL are present on fewer chromosomes with lower QTL density and protein percentage QTL are present on all but two chromosomes, most with higher QTL density (Additional data file 10). Fat percentage may be controlled by relatively fewer genes each with larger effects, whereas protein percentage may be controlled by far more genes each with smaller effects.

The milk trait QTL provide a very coarse map of genomic areas of interest that cover nearly half of the bovine assembly. Milk yield QTL overlap with 19.5% of the genomic assembly, fat yield QTL with 15.4%, protein yield QTL with 21.1%, fat percentage QTL with 12.3%, and protein percentage QTL with 33.6% of the genome assembly. The densities of genes within these QTL are very similar for each milk trait, with between 9.1 and 10.1 genes per million base pairs. Meanwhile, there are 8.4 genes per million base pairs in regions that do not overlap with any milk trait QTL. Given the gene density and number of QTL associated with each trait, each individual QTL is expected to contain between 105 and 127 genes.

To identify candidate genes within milk trait QTL, the lactation mammary gene set was intersected with the milk trait QTL. Between 12.5% and 13.7% of the genes within milk trait QTL are expressed during lactation. In other words, within a single milk trait QTL, between 13.9 and 17.1 genes are expected to be expressed during lactation. Thus, although the set of milk trait QTL reduces the search space for milk trait effectors by less than one order of magnitude, the use of expression data can contribute considerably towards the identification of candidate genes. Genes within milk trait QTL that are expressed in the mammary gland during lactation are listed in Additional data files 11-16. Milk trait effectors are likely to be near these candidate genes.

### Genome organization of milk and mammary genes

Studies of eukaryotic genomes have demonstrated that genes with coordinated expression or shared ancestry appear in clusters across the genome [20]. Given that the clustering of the casein milk protein genes is essential to their coordinated transcription in the lactating mammary gland [9,10], the arrival of the bovine genome sequence provides the opportunity to discover other gene clusters relevant to milk, lactation, or mammary biology. A genome-wide search was conducted for genomic intervals of 500 kb and greater that are statistically enriched with genes from the milk protein and mammary gene sets (see 'Genomic localization analysis' in Materials and methods). Among these gene sets, 190 non-overlapping statistically significant clusters were identified: four unique clusters in the milk protein gene set and 54, 60, 30, and 19 unique clusters in the pregnancy, lactation, involution, and mastitis mammary gene sets, respectively. Spreadsheets of all significant gene clusters are available in Additional data files 17 and 18.

The four significant milk protein gene clusters comprised the immunoglobulin genes, casein genes, fibrinogen genes, and genes that encode milk fat globule proteins. Because it is known that immunoglobulins, casein genes, and fibrinogen genes are each clustered in mammalian genomes [9,10,21,22], this is a good verification of methodology. The cluster of genes that encode milk fat globule proteins contains FASN [GenBank:NM_001012669], ARHGDIA [GenBank:NM_176650], and P4HB [GenBank:NM_174135]. However, P4HB has only been isolated in mastitic milk [11]. By manual inspection, we found that these genes also cluster in the human, mouse, and other mammalian genomes. Based on EST data, other genes in this genomic region are expressed at various times in the mammary gland. Aside from these four clusters, there does not appear to be a preponderance of putative regulatory modules among genes in the milk protein gene set. Whereas only 6.6% of the milk protein genes were within a milk protein-specific cluster, 27.9% were within one of the mammary gene set clusters. Therefore, it is likely that milk protein genes are regulated along with other mammary genes independent of the function or cellular localization of the proteins they encode.

Next, we examined whether genes were clustered according to developmental stage, but found there were no gross differences in gene clustering using this parameter. Between 24% and 30% of the genes from each mammary gene set - virgin, pregnancy, lactation, and involution - were within one of the other mammary set clusters. Likewise, 28% of the genes from the mastitis mammary gene set fell within a mammary cluster. Thus, mammary genes are not differentially clustered by developmental stage or condition.

Genes may be clustered due to shared evolution, as duplicated genes are often co-localized in the genome. In our study, a significant cluster required a minimum of three genes that were not paralogs. When the paralog requirement was removed, only seven additional unique clusters of triplets or greater were identified. Significant clusters with more than one paralog appear to be confined to the major histocompatibility complex region on bovine chromosome 23. These data suggest that recent duplication is not a common driver of clustered mammary genes in the bovine genome.

In summary, the milk protein genes generally do not form clusters with each other but do appear to form clusters with other mammary genes. Milk protein genes may be regulated along with other lactation genes without regards to the final destination of the gene product. As mammary genes are generally clustered neither by developmental stage nor due to recent duplication, it appears that the need for co-expression in the mammary gland is the denominator for co-localization rather than co-functionality or shared ancestry. This organization in clusters of co-expressed mammary genes might be constrained by unidentified distal *cis*-acting elements [20], chromatin conformation [23], or coordinately expressed micro-RNAs [24].

### Milk and mammary gene copy number trends in mammals

**Figure 3:**
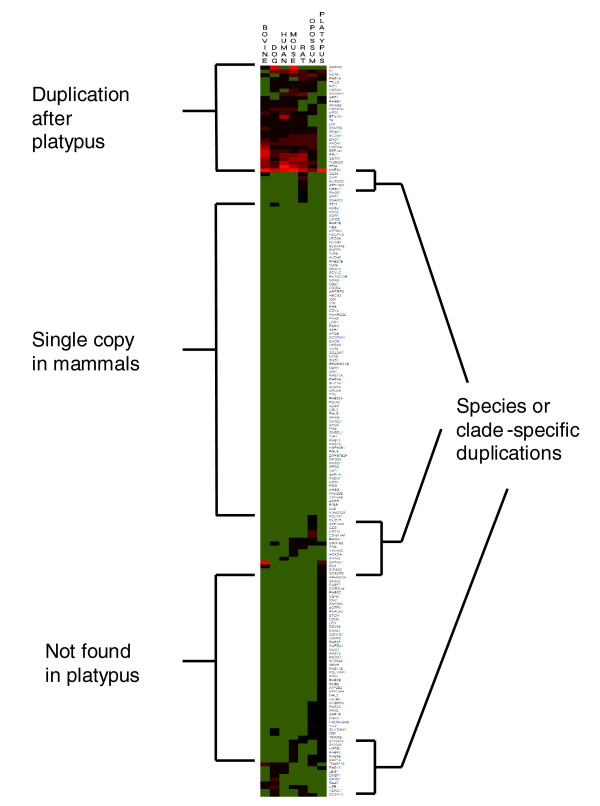
Heatmap of milk protein gene copy numbers across mammals. Milk protein genes were clustered by copy number using the K-means algorithm followed by seriation within each cluster. Major trends, which convey the consensus profile of the cluster, are delineated by brackets. Most milk protein genes are either present as a single copy in each mammalian genome or as a single copy in all therian genomes. Duplicated genes are expanded after platypus in either a general or a species- or clade-specific manner. Black squares indicate that the gene was not found in a particular species, yellow-green squares indicate a single copy of the gene, and red squares indicate two or more copies of the gene. Brighter red squares indicate higher copy numbers.

Gene copy number contributes to genetic diversity both between and within species. Here, copy numbers of bovine milk protein genes were determined in the bovine, human, mouse, rat, dog, opossum, and platypus genomes using orthologs generated for all bovine consensus gene models (see 'Orthology delineation' in Materials and methods). Genes from the milk protein gene set that were uniquely duplicated in *B. taurus *and those that were missing copies in one or more of the placental mammals were manually curated (see 'Curation of milk protein orthologs' in Materials and methods). K-means clustering of these curated milk protein gene orthologs followed by seriation within each cluster yielded the heatmap shown in Figure 3. Three major trends were identified: single copy of the gene across Mammalia; gene not found in platypus; and duplication after platypus.

The absence of a milk or mammary gene in platypus or duplication after platypus (Figure 3) may be due to the expansion of gene families in the common therian ancestor. However, some of these genes may not be truly missing in the platypus genome, but may be undetectable by our methods due to incomplete or incorrect assembly of the platypus genome, lower sequence identity, or the inherent bias created by defining milk and mammary genes in the bovine genome. The identification of platypus orthologs of other genes in the bovine genome would also be affected by these biases; therefore, we next compared milk and mammary gene copy number trends to those genome-wide.

For each major trend shown in Figure 3, rates of occurrence among the uncurated orthologs of the milk protein and lactation mammary gene sets were compared with the orthologs of all bovine consensus gene models using a hypergeometric distribution to determine statistical significance. More bovine milk protein orthologs were found in all six studied mammalian genomes than would be expected given the rate at which other bovine orthologs were found in these genomes (*P *< 0.0001). Genes expressed during bovine lactation were also more likely than other genes to have orthologs in all of the mammalian genomes (*P *< 0.0001). In other words, milk and mammary genes are more likely than other genes to be found in all mammals. This result might be explained in part by an increased power to detect more conserved genes (see 'Conservation of milk and mammary genes in mammals' below). There were also statistically fewer lactation genes missing in the platypus (*P *< 0.005) and opossum genomes (*P *< 2.2 × 10^-20^); however, the number of milk protein genes missing in these genomes did not differ from the genome-wide rate. Finally, more milk protein and lactation genes were duplicated after platypus compared with the whole genome (*P *< 0.001 and *P *< 0.03, respectively). Together, these data support the essentiality of milk and mammary genes in Mammalia as well as suggest the possibility for expanded functionality in marsupials and placental mammals.

Milk protein gene copy number variation may potentially contribute to the diversity of milk composition. Ortholog analysis indicated that the gene for beta-lactoglobulin (LGB), one of the most abundant proteins in milk, is duplicated in the dog and bovine genomes (Figure 3). In the bovine genome, this gene is located at the position of a previously predicted pseudogene [25]. It has similarity to LGB-II genes in the horse and cat [26-29]. The similarity of this second gene to LGB-II in the horse, cat, and dog suggests that the LGB duplication existed in the common ancestor of the laurasiathians (Figure 1). Using two different primer pairs, we were unable to identify the LGB-II transcript in bovine mammary tissue samples using RT-PCR (see Additional data file 22 for details). It is likely that the duplicated LGB gene is not expressed in the bovine mammary gland and that the presence of this duplication does not influence the concentration of LGB in bovine milk.

LGB is apparently not present in human or mouse milk [30], although LGB-like proteins have been isolated from the milk of other primates [31-33]. A human protein, progestagen-associated endometrial protein (PAEP), has significant homology to the bovine and equine LGB-II-like genes [29,34-36]. Although PAEP expression has been detected in the epithelial cells of human breast tissue [37], neither its presence nor that of an apparent LGB-like pseudogene [GenBank:AH011480] that flanks the PAEP gene [GenBank:NM_001018049] has been verified in human milk. We found that the LGB-like and PAEP genes are flanked by GLT6D1 [GenBank:NM_182974] and OBP2A [GenBank:NM_014582] in both the human and bovine genomes. This observation, combined with the fact that the baboon has both a PAEP gene [38] and a LGB gene [33], suggests that the primate genes arose by duplication of an ancestral gene before the Laurasiatheria and Eurochontoglires diverged. We were unable to find this region in the rodent or rabbit genomes, and an evolutionary break point is present in mouse and rat in this region [39], suggesting that these genes may have been lost after the split between primates and glires. Although the presence of LGB in laurasiathian milk and its absence in rodent milks has an obvious genetic basis, we cannot yet explain the absence of LGB in human milk.

Some immune components of milk are uniquely duplicated in certain species or clades. For example, SAA3 [GenBank:NM_181016], which is duplicated in the bovine and dog genomes (Figure 3), is thought to be involved in mucin induction in the gut [40,41] and a human analog, SAA1, functions as an opsonin for Gram-negative bacteria [42]. The Cathelicidin gene family is greatly expanded in the bovine, opossum, and platypus genomes, with 10, 8, and 12 copies, respectively [43-45], but some of the opossum and platypus orthologs were not found in our automated analysis due to their high heterogeneity. Expansions in this gene family may reflect increased exposure to bacteria at epithelial surfaces in these species. Our results show that the CD36 gene [GenBank:NM_174010], which encodes a scavenger receptor, has duplications in the *B. taurus *and rat genomes. Beta-2-microglobulin [GenBank:NM_173893] has a second copy in the bovine genome and may also have a duplicate in the platypus genome. This gene encodes one of two chains in the IgG transporter FcRn, which transfers IgG molecules across epithelial cells [46]. Other variations in milk protein gene copy number (Figure 3) potentially give rise to diversity in milk protein composition.

Milk protein gene loss does not appear to be a common occurrence. Of the bovine milk protein genes with an ortholog identified in the platypus genome (Figure 3), all but ten genes were found in all of the other studied mammalian genomes. However, because the bovine milk proteome is used as the reference, the loss of some milk protein genes in placental mammals relative to the monotreme and marsupial mammals may have been missed in our analysis. For example, whey acidic protein has been identified in the milk of many mammals such as mouse, rat, opossum, and platypus, but it is absent in bovine milk due to a frameshift mutation in the whey acidic protein gene [47]. A full proteomic analysis of the milk samples from extant monotremes and marsupials will be needed to identify gene loss in placental mammals.

Our analysis of milk protein gene copy numbers has several other limitations. First, the mammalian orthologs of bovine consensus gene models derived on a genome-wide basis (see 'Orthology delineation' in Materials and methods) may be inaccurate for genes in which the bovine gene model is incorrect or may be incomplete when orthologs are too divergent to be detected by this method. Although we attempted to overcome these limitations by manually curating milk protein gene orthologs, the analysis is only as good as the available genome sequences, and some duplications and deletions may have been missed due to errors and gaps in the genome assemblies. Directed sequencing will be needed to confirm specific results. However, we can generally conclude that there is considerable copy number variation of milk protein genes that may contribute to the taxonomic diversity of milk composition.

### Taxonomic relationships of the milk protein genes

**Figure 4:**
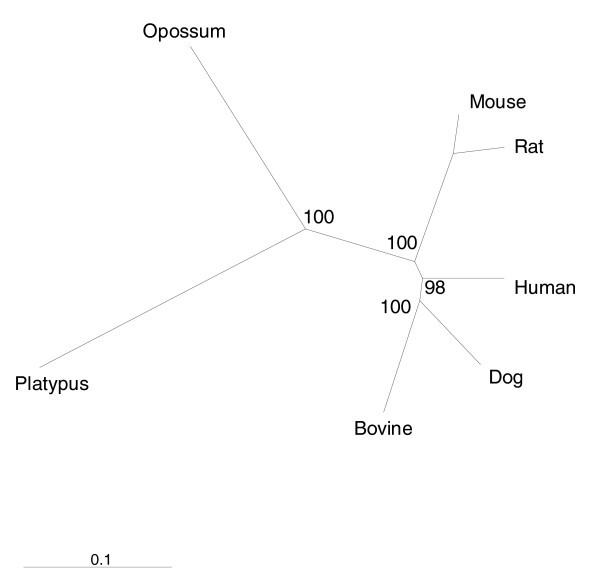
Relationships between the milk protein sequences of mammalian taxa. This milk protein consensus tree, which is incongruous with the accepted phylogeny shown in Figure 1, was derived from a super-alignment of milk protein amino acid sequences for those genes with single copy orthologs in all seven species. The numbers indicate the percent of bootstraps that support the internal branch and the length of the scale bar represents the number of amino acid substitutions per unit site.

**Figure 5:**
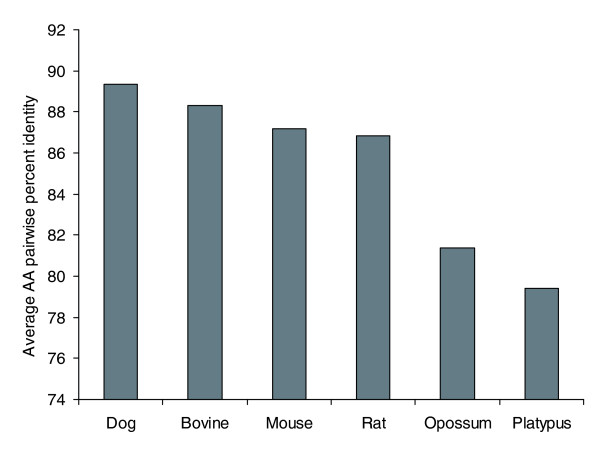
Pairwise percent identity of human milk proteins with milk proteins of other species. Bars depict the average amino acid (AA) pairwise percent identity between human milk proteins and those of the species named on the x-axis. Note that human milk proteins are more similar to those of dog and bovine than to rodents and the other species depicted.

To understand the relationships of the milk proteins between mammalian taxa, a consensus tree of those milk proteins with single copy orthologs in the human, mouse, rat, dog, bovine, opossum, and platypus genomes was constructed using a super-alignment of the concatenated sequences (see 'Consensus tree construction' in Materials and methods). An unrooted radial tree depicting the relationships of the milk protein sequences (Figure 4) differs from the accepted phylogeny (Figure 1). Rodent milk proteins are more divergent from human milk proteins than are dog and bovine milk proteins despite the fact that the rodent and human common ancestor is more recent. To further test the relationships of human milk proteins with those of other taxa, pairwise percent identity (PID) was calculated between the human protein and its putative ortholog for the set of single copy orthologs present in all seven taxa. Average pairwise PIDs for the milk protein gene set confirm that human milk proteins are closest to dog, followed by bovine, then the rodents, then opossum and platypus (Figure 5). This observation is not unique to milk proteins as it is also true on a genome-wide basis [43]. It has been proposed that rodent proteins are more divergent from human than are bovine proteins because rodents have a faster reproductive rate and are, therefore, evolving more quickly [43]. Although rodent milk proteins may appear more distant from human milk proteins than are bovine milk proteins, whether these differences have functional importance is a matter for future scientific inquiry.

### Conservation of milk and mammary genes in mammals

**Figure 6:**
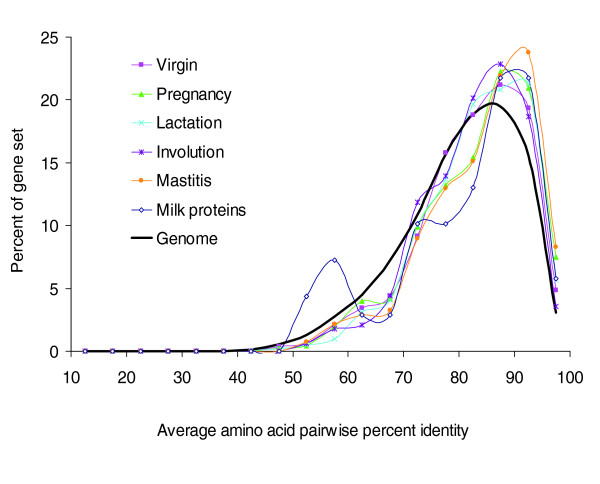
Average pairwise percent identities of milk and mammary genes across mammals. The distribution of average amino acid pairwise PID of amino acid sequences across the seven taxa - human, mouse, rat, bovine, dog, opossum, and platypus - is plotted for those genes in the virgin, pregnancy, lactation, involution, and mastitis mammary gene sets, the milk protein gene set, and all bovine consensus genes. Only genes with a single copy in each of the seven genomes were used for the analysis. Milk and mammary genes are more conserved across mammals than other genes in the genome.

To determine whether milk and lactation-related genes are more or less conserved across mammals than other genes, average PIDs of the 21 pairwise comparisons of the seven taxa were computed on a genome-wide basis for all bovine consensus gene models and genes from the milk protein and mammary gene sets with single copy orthologs in these taxa (Figure 6). The distribution of the average pairwise PIDs of the milk protein gene set did not significantly differ from the whole genome distribution, nor did the means of the two distributions significantly differ (see 'Statistical analysis of PID distributions' in Materials and methods). However, when the sample size was increased by individually assessing pairwise PIDs between human and each of the seven taxa, requiring in each case that orthologs be single copies only in bovine and the two taxa being compared, milk protein sequences were statistically more conserved between human and other mammals than the products of other genes in the genome (see Additional data file 20 for details). The human-bovine distribution is most dramatically different from the whole genome as a full quarter of the set of the 137 milk protein genes with single copies in these two genomes are very highly conserved with a pairwise PID of 97.5% or greater.

Of the average pairwise PID distributions of the mammary gene sets in Figure 6, all are significantly different from the genome-wide distribution. The means of their distributions also differ from the genome-wide mean. As a group, mammary genes of every developmental stage and condition appear to be more conserved across Mammalia, on average, than other genes in the genome.

To discover which milk proteins are most conserved in mammals, the average pairwise PIDs among the seven mammalian taxa were computed for all genes from the milk protein gene set with single copy orthologs in the manually curated set (see 'Curation of milk protein orthologs' in Materials and methods). The top 25 most conserved milk proteins across all seven mammals are listed in Table 2. These proteins have greater than 95% identity across mammals, some more than 99%, despite the fact that they have not shared a common ancestor for more than 160 million years. Based on the amino acid length and conservation, we can predict that these milk proteins have a small size with functions that depend on strictly conserved structure.

**Table 2:** Highly conserved milk proteins

Protein name	GLEAN ID*	Gene symbol	Average PID
Rab 11A	GLEAN_20537	*RAB11A*	100
GTP binding protein G I G S G T beta subunit 1	GLEAN_21827	*GBB1*	100
GTP binding regulatory protein beta 2 chain	GLEAN_22534	*GNB2*	100
Rho C	GLEAN_13128	*RHOC*	99.8
Rab 11B	GLEAN_03051	*RAB11B*	99.6
Rap 1b	GLEAN_10112	*RAP1B*	99.6
GTP binding protein Sara	GLEAN_16602	*SAR1A*	99.5
Rab 3A	GLEAN_02763	*RAB3A*	99.5
Histone 2, H2ab	GLEAN_23583	*HIST2H2AB*	99.2
SAR1B protein	GLEAN_08536	*SAR1B*	98.9
14-3-3 protein beta alpha	GLEAN_04527	*YWHAB*	98.9
Leucine-rich repeat containing protein 8	GLEAN_26477	*LRC8A*	98.8
Rab 18	GLEAN_21462	*RAB18*	98.6
Rho GDP-dissociation	GLEAN_11194	*ARHGDIA*	98.5
Rab 5C	GLEAN_17381	*RAB5C*	98.5
AD158	GLEAN_08550	*LRC8C*	98.1
Rab 3C	GLEAN_20950_P10949	*RAB3C*	98.0
Eukaryotic translation initiation factor 4, gamma 2	GLEAN_19671	*EIF4G2*	97.9
ATP synthase, H+ transporting, mitochondrial F1 complex, alpha subunit	GLEAN_03119	*ATP5A1*	97.7
ARP3 (actin-related protein 3, yeast) homolog	GLEAN_25161	*ACTR3*	97.5
Vimentin	GLEAN_20783	*VIM*	97.3
GTP binding protein alpha 14	GLEAN_08781	*GNA14*	97.2
Endoplasmin precursor (GRP94/GP96)	GLEAN_20794	*HSP90B1*	97.1
Lymphocyte cytosolic protein 1 (65 K macrophage protein/L-plastin)	GLEAN_05236	*LCP1*	97.0
Retinal short-chain dehydrogenase/reductase	GLEAN_03662	*SDR1*	96.9

*Manually curated full length sequences are indicated by the following accession format: GLEAN_ID_ACCESSION where ACCESSION is the UniProt accession for the replacement amino acid sequence and GLEAN_ID is the bovine consensus gene identifier for the original sequence.

Nearly all of the highly conserved milk proteins (Table 2) are found in the milk fat globule membrane proteome. GO analysis of these proteins yields four enriched terms: 'GTPase activity,' 'GTP binding,' 'small GTPase mediated signal transduction,' and 'intracellular protein transport.' Twelve of the proteins listed in Table 2 are annotated with one or more of these GO terms. GTPases are known to be involved in the exocytotic pathway by which proteins are trafficked from the Golgi compartment to the plasma membrane. Further, GBB1 [GenBank:NM_175777], RAB11B [GenBank:NM_001035391], RAP1B [GenBank:NM_175824], YWHAB [GenBank:NM_174794], and RAB18 [GenBank:NM_001075499] listed in Table 2 have previously been isolated in Golgi fractions from the mammary glands of pregnant and lactating rats [48]. An additional four milk proteins, SAR1A [GenBank:NM_001034521], SAR1B [GenBank:NM_001035315], RAB3A [GenBank:NM_174446], and RAB3C [GenBank:NM_001046606], are annotated with the GO term 'secretory pathway.' The finding that so many of these secretion-related proteins are associated with the milk fat globule membrane suggests they may also be involved in the highly specialized process by which the milk fat globule is secreted or that the exocytotic and lipid secretion pathways intersect at some point during the secretion process. Because the conserved proteins listed in Table 2 are related to the generic molecular function of secretion, it seems highly likely that they facilitate the secretion of milk lipid.

Conservation of mammary genes relative to other genes in the genome suggests hypotheses about the evolution of milk production. First, conservation of mammary genes involved in all developmental stages supports the hypothesis that, at the genetic level, the basic biological transformation of the virgin gland through pregnancy, lactation, and involution is conserved among all mammals, and occurred by co-opting existing structures and developmental pathways. Second, many of the most highly conserved proteins found in milk are constituents of the milk fat globule membrane and are known to be part of the secretory process. High conservation of these genes between platypus, opossum, and the placental mammals indicates that molecular mechanisms of secretion were already in place 160 million years ago.

### Divergent milk protein genes in mammals

Because the technique for ortholog detection relies on a minimum threshold of conservation, orthologs of many of the more divergent proteins could not be found in the platypus or opossum genomes. Therefore, to determine which proteins in milk are most divergent in mammals, average PIDs were computed across only the five placental mammals. The 25 most divergent milk proteins across placental mammals are presented in Table 3. These milk proteins are primarily secreted or cell-surface proteins with structures that are apparently not constrained by function relative to other proteins in milk. Four GO terms associated with these proteins are enriched: 'pattern binding,' 'response to other organism,' 'inflammatory response,' and 'extracellular space.'

**Table 3:** Highly divergent milk proteins

Protein name	GLEAN ID*	Gene symbol	Average PID
Alpha-S1-casein	GLEAN_22124_P02662	*CSN1S1*	44.4
G protein Xlalphas	GLEAN_10239	*GNAS*	48.6
Kappa casein	GLEAN_22128	*CSN3*	51.3
Mucin 1	GLEAN_00552	*MUC1*	52.2
Beta casein	GLEAN_22133	*CSN2*	55.4
Rab 10	GLEAN_18819_A6QLS9	*RAB10*	57.6
SCAMP2	GLEAN_09359_A6QR35	*SCAMP2*	63.6
Fetuin	GLEAN_07528	*AHSG*	64.5
Immunoglobulin IgM	GLEAN_18189	*IgM*	65.0
Polymeric-immunoglobulin receptor precursor	GLEAN_25657	*PIGR*	66.7
Keratin 9	GLEAN_21315	*KRT9*	66.9
CD14	GLEAN_04279	*CD14*	68.0
Rab 7	GLEAN_25742	*RAB7A*	68.3
Peptidoglycan recognition protein	GLEAN_12036	*PGLYRP1*	68.5
Fibrinogen alpha chain	GLEAN_24372	*FGA*	68.6
Apolipoprotein B	GLEAN_00959	*APOB*	69.4
Apolipoprotein E	GLEAN_10715	*APOE*	70.0
Glycoprotein antigen MGP57/53 (lactadherin/bP47 protein)	GLEAN_17418	*MFGE8*	70.3
Toll-like receptor 4	GLEAN_05263	*TLR4*	70.7
Alpha-lactalbumin	GLEAN_17221	*LALBA*	70.7
MUC15 protein	GLEAN_04480	*MUC15*	71.0
Macrophage scavenger receptor types I and II	GLEAN_13926	*MSR1*	72.1
Toll-like receptor 2	GLEAN_24366	*TLR2*	73.1
Albumin (precursor)	GLEAN_11814	*ALB*	74.6
Chitinase-like protein 1 (CLP-1)	GLEAN_07846	*CHI3L1*	76.3

*Manually curated full length sequences are indicated by the following accession format: GLEAN_ID_ACCESSION where ACCESSION is the UniProt accession for the replacement amino acid sequence and GLEAN_ID is the bovine consensus gene identifier for the original sequence.

The greatest inter-species divergence among milk protein sequences occurs with those proteins that are most abundant in milk (caseins, alpha-lactalbumin (LALBA)), those most abundant in plasma (fetuin, albumin), and with those contributing to immunity. The casein proteins are the most divergent of the milk proteins, with an average pairwise PID of only 44-55% across placental mammals. Nutritionally, the caseins provide the suckling neonate with a source of amino acids and with highly bioavailable calcium. Additionally, peptides derived from partially digested caseins have potential anti-microbial, immune-modulating, and other bioactive properties. The fact that the caseins are the most divergent of the milk proteins suggests that the nutritional and immunological functions of these proteins do not particularly constrain their amino acid sequence and structure.

The sequence divergence of LALBA is surprising given its essentiality to the synthesis of lactose, the primary source of digestible carbohydrate. *LALBA *encodes a protein that forms the regulatory subunit of the lactose synthase heterodimer. However, additional functions of LALBA have emerged. When human LALBA is partially unfolded and bound to oleic acid, it functions as an apoptotic factor that kills tumor cells and immature cells, but not healthy differentiated cells [49]. Thus, it is possible that this variant of LALBA protects the gut of the human neonate. Furthermore, the apoptotic capabilities of LALBA appear to be utilized in the regulation of involution of the mammary gland. A recent study suggests that Cape fur seals escape apoptosis and involution of the mammary gland during long foraging trips because they lack the LALBA protein [50]. While lactose synthesis may be a common essential function, it appears that it does not overly constrain the sequence divergence of LALBA. The sequence divergence of LALBA may rather be related to the potential of this protein to modulate species-specific strategies related to immune function and the regulation of the mammary gland.

The most divergent immune-related proteins in milk are products of the following genes: *mucin 1 *(*MUC1*) [GenBank:NM_174115], *immunoglobulin IgM *[GenBank:BC114809], *polymeric-immunoglobulin receptor *(*PIGR*) [GenBank:NM_174143], *peptidoglycan recognition protein *(*PGLYRP1*) [GenBank:NM_174573], *CD14 *[GenBank:NM_174008], *Toll-like receptor 2 *(*TLR2*) [GenBank:NM_174197], *Toll-like receptor 4 *(*TLR4*) [GenBank:NM_174198], *macrophage scavenger receptor types I and II *(*MSR1*) [GenBank:NM_001113240], and *chitinase-like protein 1 *(*CHI3L1*) [GenBank:NM_001080219]. In milk, CD14 and TLR2 are present in soluble forms and may neutralize pathogens by binding to them as decoy receptors [13,14]. MUC1 prevents the binding of pathogenic bacteria to epithelial cells *in vitro *(R.L. Tellam, personal communication). Our finding that the most divergent milk protein genes are those that confer immunity presumably reflects a flexibility to confront a wide variety of pathogen challenges.

### Evolution of milk and mammary genes along the bovine lineage

To investigate the selective constraints on the evolution of bovine milk and mammary genes, the rate of non-synonymous substitutions per non-synonymous site (d_N_) to synonymous substitutions per synonymous site (d_S_) was estimated for proteins in each gene set using bovine genes and their putative orthologs in the human and mouse genomes (see 'Evolutionary analysis along the bovine lineage' in Materials and methods for details). The average d_N_/d_S _ratio of the genes from the milk protein and mammary gene sets (Table 4) was significantly below the genome average (Mann-Whitney U test, *P *< 0.05), indicating that milk and mammary genes are subject to more stringent selective constraint than other genes in the bovine genome.

**Table 4:** Milk and mammary gene average d_N_/d_S_

Gene set	Number of genes with computed d_N_/d_S_	Average d_N_/d_S_
Milk protein	149	0.13
Virgin mammary	3,091	0.13
Pregnancy mammary	1,032	0.12
Lactation mammary	2,477	0.12
Involution mammary	704	0.12
Mastitis mammary	615	0.12
Whole genome	14,354	0.16

Given the taxonomic diversity of milk composition, we expected that the processes of lactation would be under stronger selective pressure than the genes that give rise to proteins in milk. However, the average d_N_/d_S _of the milk protein gene set was similar to that of the lactation mammary gene set (Table 4). This result suggests that species-specific variation in milk composition is primarily due to mechanisms other than milk and mammary protein sequence variation.

Next, milk and mammary genes were evaluated for positive selection. A gene is inferred to be subject to positive selection when d_N_/d_S _is significantly greater than 1. Of the 6,530 genes from the milk protein and mammary gene sets, only two bovine genes with d_N_/d_S _>1 were significant under the likelihood ratio test (see 'Evolution analysis under the bovine lineage' in Materials and methods): *ADP-ribosyltransferase 4 *(*ART4*) [GenBank:AJ291442] and *prenylcysteine oxidase 1 *(*PCYOX1*) [GenBank:NM_001105474]. The *ART4 *gene product, which has previously been reported to be subject to positive selection in cattle [51], is an erythrocyte protein that carries antigens to the Dombrock blood group. *PCYOX1 *produces a protein that degrades a variety of prenylcysteines. Using RT-PCR to determine *PCYOX1 *and *ART4 *mRNA levels in alveolar mammary tissue from virgin, prepartum, lactating, involuting and dried-off cows (Additional data file 22), we found that *PCYOX1 *and *ART4 *are not differentially expressed in these tissues. The accelerated evolution of these genes may be unrelated to mammary biology.

Two abundant milk protein genes, *beta-casein *(*CSN2*) [GenBank:NM_181008] and *kappa-casein *(*CSN3*) [GenBank:NM_174294], were among those with d_N_/d_S _>1, but they were not statistically significant under the likelihood ratio test (see 'Evolution analysis along the bovine lineage' in Materials and methods). The requirement that the entire gene shows statistical evidence of positive selection may be too stringent. Evidence of positive selection within the family Bovidae has been previously detected in a 34-codon region of *CSN3 *[52]. Further site-specific evolutionary analysis of the casein genes may be warranted.

Despite the domestication of cattle for milk production, breeding regimes have not caused the apparent accelerated evolution of even a single milk protein or member of the lactation mammary gene set. Furthermore, milk and mammary genes are undergoing stronger purifying selection than other genes in the bovine genome. It has previously been theorized that the evolution of the mammary gland has been subject to forces that maximize the survival of the mother-child pair [53]. Because all components in the milk are produced at the expense of the mother, it can be argued that few superfluous components would survive evolution. Our findings are consistent with this hypothesis. Genes encoding milk components and other genes expressed in the mammary gland were found to be under significant negative selection compared to the whole genome, highlighting the essentiality of milk in mammalian evolution.

## Conclusions

The availability of the *B. taurus *genome sequence assembly marks the beginning of a new era for the study of milk and mammary biology. Using this assembly, we identified 197 unique milk protein genes and over 6,000 mammary-related genes distributed across all bovine chromosomes. Intersecting these genes with 238 curated milk-trait QTL, we reduced the search space for milk trait effectors by more than an order of magnitude.

Comparing genes of seven mammalian species, we found that more milk and mammary genes are present in all mammals and more were duplicated after the common ancestor with platypus than other genes in the bovine genome. We also found that, on average, milk and mammary genes are more conserved than other genes among mammals and are evolving more slowly than other genes in the bovine genome. The higher conservation of these genes, as well as the strong negative selection and absence of positive selection, supports the hypothesis that the evolution of milk has been constrained to maximize the survival of both mother and offspring.

Our findings also suggest that the species-specific variation in milk composition is primarily due to mechanisms other than protein sequence variation. Variation in copy number of the milk protein genes may contribute to the taxonomic diversity of milk composition, as exemplified by duplications of some immune-related milk proteins. Additional mechanisms, such as transcriptional and translational regulation of genes expressed in the mammary gland as well as other organs involved in energy partitioning may be larger contributors to milk composition variation. In future studies, non-coding regions of the genome, particularly those with putative regulatory function, will need to be explored as potential sources of species-specific variation in milk composition.

We found that mammary genes are co-localized in the bovine genome, implying co-regulation of expression in mammary epithelial cells. Our study of the most complete milk proteome to date demonstrates that milk proteins do not generally cluster with each other, but do cluster with mammary genes, and therefore are likely co-regulated. The casein proteins are therefore unique among the milk protein genes in both their divergence between species and in their genomic organization.

Across the seven mammalian genomes, we found that the most divergent milk proteins are known to have nutritional and immunological properties, whereas the most conserved are within the milk fat globule proteome. The high conservation of genes apparently involved in milk fat globule secretion suggests that the cellular anatomy of secretion may be conserved across species and likely shared among secretory organs. Likewise, the higher conservation of mammary genes, compared with other genes, suggests that the ontogeny of the mammary gland occurred by co-opting existing structures and developmental pathways. Lactation may be less than 200 million years old, but its biological roots are far more ancient.

## Materials and methods

### Collection of the milk protein gene set

Amino acid sequences corresponding to the protein identifiers reported in two proteomic studies [11,12] were collected from SwissProt, UniRef, TREMBL, and NCBI databases. A custom track of the bovine consensus gene models [43], or 'GLEAN' models, was created and uploaded to the University of California Santa Cruz (UCSC) Genome Browser [54]. The milk protein amino acid sequences were aligned against the bovine genome draft 3.1, also known as 'Aug. 2006,' using BLAT [55]. The best scoring hits were manually reviewed against the custom GLEAN track in the UCSC Genome Browser. From the protein sequence identifiers in the Smolenski and Reinhardt studies [11,12], 189 unique milk proteins were mapped to GLEAN IDs. The GLEAN IDs corresponding to the six most abundant milk proteins, alpha-S1-casein, alpha-S2-casein, beta-casein, kappa-casein, LGB, and LALBA, were also added to this gene set. Additionally, a script was written to identify genes in the bovine annotation database annotated by the lactation group that did not already exist in our milk protein gene set. Those genes were then manually screened for proteins known to be in milk. Two additional proteins were identified: lactotransferrin and secreted phosphoprotein 1 (osteopontin). In total, the milk protein gene set consists of 197 unique genes that encode proteins known to be in bovine milk (Additional data files 1 and 2).

### Collection of the mammary gene sets

Bovine mammary gland EST libraries available through the NCBI EST database [56] were surveyed to define condition- or developmental stage-specific mammary gene sets. Descriptions of the EST libraries used as well as reasons for exclusion of other EST libraries are given in Additional data file 21. The EST libraries used include the BMGA, BMLB, BMPA, BMUA, and BMVB libraries developed by AgResearch Ltd and Genesis Ltd in New Zealand as well as the FNM and FLM libraries developed by ViaLactia Biosciences Ltd in New Zealand. Custom tracks for the UCSC Genome and Table Browsers [54,57] were created for all GLEAN models from the bovine genome project [43] and for all of these EST libraries. The intersection filter of the UCSC Table Browser was used to identify the GLEAN models that overlapped with ESTs in these libraries. Mammary gene sets were defined as follows: virgin = (BMVB or FNM); pregnancy = BMPA; lactation = (BMGA or FLM); involution = BMLB; and mastitis = BMUA. In total, these mammary gene sets contain 6,469 unique genes derived from over 94,000 ESTs. Gene identifiers for the genes that comprise each mammary gene set are given in the spreadsheets of Additional data file 1. UCSC custom tracks of these genes sets are provided in Additional data files 3-7.

### Gene Ontology analysis

Bovine genes from the milk protein and mammary gene sets were mapped to human orthologs using a three-way reciprocal best BLAST hit approach between human, mouse, and bovine as implemented in the 'Ortholuge' program [58]. Using GO-Elite version 1.17 beta [59], the human orthologs of milk and mammary genes were analyzed for over-represented GO terms compared with the full set of human orthologs. GO-Elite calculates a Fisher's exact test z-score for unique genes corresponding to a GO term from the user's input list compared with the reference list. To calculate a *P*-value for each GO term, GO-Elite randomly selects the same number of input genes or probe sets in the user's input gene list from the reference list 2,000 times to determine the likelihood of obtaining a z-score greater than or equal to the empirically derived z-score. To adjust for multiple hypothesis testing, the Benjamini-Hochberg correction is used to calculate an adjusted *P*-value. GO-Elite determines the set of minimally redundant significant GO terms by examining the scores and relative positions of all high-scoring terms within the hierarchy to select those terms that maximally describe the biology without removing valuable information. GO terms with an adjusted *P*-value ≤ 0.05 were taken to be significant.

### Pathway analysis

Ingenuity Pathways Analysis [60] was used to identify metabolic and signaling pathways that are over-represented by the human orthologs of milk and lactation-related bovine genes compared with the human orthologs of all of the genes from the bovine consensus gene model (GLEAN) set. The Ingenuity Pathways Analysis library of canonical pathways includes 80 metabolic and 72 signaling pathways that have been incorporated from various resources and hand-curated. A Fischer's exact test was used to calculate a *P*-value to determine the probability that the enrichment of the canonical pathway with the gene set of interest is explained by chance alone. In this paper, this *P*-value is referred to as the unadjusted *P*-value. Enriched pathways with an unadjusted *P*-value ≤ 0.05 and associated with at least three genes of interest are referred to as marginally significant. To improve the stringency of the test, a Benjamini and Hochberg multiple testing correction was applied to the unadjusted *P*-values with a custom R script using the multtest library [61,62]. Pathways reported to be statistically significant were those with a Benjamini and Hochberg adjusted *P*-value ≤ 0.05.

### Genomic localization analysis

The bovine genome was explored for clusters of milk and lactation-related genes that are more proximal to each other in the genome than would be expected by chance. Using the method developed by Salomonis *et al*. [63], 500 kb windows on the genome that start in the same position as a gene were tested as candidate co-location clusters. For each such candidate cluster, a list of genes that overlap the window was assembled. Strand information was ignored, so that a gene was considered to start at its lowest coordinate. Genomic sequence not assembled to chromosomes was excluded.

For each of the milk protein and mammary gene sets, the statistically expected number of genes from the cluster in the gene set was calculated, given the number of genes on chromosomes both inside and outside of the gene set and using a hypergeometric distribution. Genes were considered to comprise a co-location cluster if there were at least three non-paralogous genes of interest in the region and the number of candidate cluster genes from the gene sets was significantly higher than chance (*P *≤ 0.05). A multiple test correction against all the clusters was performed with a Benjamini and Hochberg false discovery rate [61,62]. When multiple clusters contained the same genes or subsets of genes, the cluster with the lowest *P*-value was reported.

### Orthology delineation

Orthologs of the milk and mammary genes were filtered from the whole genome ortholog set [43]. Briefly, groups of orthologous genes were automatically identified using a previously employed strategy [64] that is based on all-against-all protein sequence comparisons using the Smith-Waterman algorithm, followed by clustering of best reciprocal hits from highest scoring ones to 10^-3 ^e-value cutoff for triangulating best reciprocal hits or 10^-6 ^cutoff for unsupported best reciprocal hits, and requiring a sequence alignment overlap of at least 30 amino acids across all members of a group. Furthermore, the orthologous groups were expanded by genes that are more similar to each other within a proteome than to any gene in any of the other species, and by very similar copies that share over 97% sequence identity. The procedure was applied to the initial bovine set of consensus gene models [43] and to the longest transcript per gene of the Ensembl v.45 [65] gene sets of human, mouse, rat, dog, opossum, and platypus.

### Curation of milk protein orthologs

Orthologs of milk protein genes (see 'Orthology delineation' above) were curated manually if they were uniquely duplicated in *B. taurus *or missing copies in one or more of the placental mammals. Fifteen genes uniquely duplicated in *B. taurus *were removed from the curated ortholog set for the following reasons: four were pseudogenes - gene duplicates without introns; four were not assembled on a chromosome and, therefore, likely to represent reading of the same sequence; and seven were a result of an assembly error or poor GLEAN prediction. The pseudogenes identified were those of PFN1 (GLEAN_02143), RAB18 (GLEAN_21462), RAP1B (GLEAN_10112), and YWHAZ (GLEAN_11922). A perfect duplicate of GAPDH (GLEAN_13969) that exists in both Btau 3.1 and 4.0 assemblies was retained, but further research is needed to determine if it is a true expansion. To find missing orthologs of milk protein genes in placental mammals, the Ensembl databases of those species with orthologs not found by automated detection were searched using BLAST and the bovine consensus gene and protein sequences. Additional known orthologs were collected from UniProt. In total, 15 erroneous duplicate genes and 37 missing orthologs were identified through manual curation. This curated ortholog set is available in Additional data file 19.

For conservation analyses, amino acid sequences of bovine genes annotated as erroneous in the bovine genome annotation database [66] were replaced with manually curated full-length sequences from UniProt [67]. Replaced sequences are indicated by the following accession format in Tables 2 and 3: GLEAN_ID_ACCESSION where ACCESSION is the UniProt accession for the replacement amino acid sequence and GLEAN_ID is the bovine gene model identifier for the original sequence.

### Milk protein gene copy clustering and visualization

Perl and shell scripts were written to create input files for the clustering tool, Cluster version 2.20 [68]. A K-means clustering algorithm was applied in Cluster, and the resulting clusters were seriated using a multiple-fragment heuristic in PermutMatrix [69]. Whether or not clustering was applied, all heatmaps were seriated and visualized in PermutMatrix.

### Consensus tree construction

Single copy ortholog identifiers of milk protein genes were extracted from the whole genome set of orthologous clusters (see 'Orthology delineation' in Materials and methods). Amino acid sequences for these identifiers were downloaded from the Ensembl database release 45 [70]. Multiple alignments of the milk proteins were constructed using MUSCLE [71]. The multiple alignments were then concatenated into a super-alignment that was used to create a maximum likelihood consensus tree with PhyML [72,73]. The tree was constructed based on the JTT model of amino acid sequence evolution [74] with rates assumed to vary among sites according to a gamma distribution. Support for the tree's nodes is given by 100 bootstraps.

### Statistical analysis of PID distributions

A Wilcoxon rank sum test with continuity correction (Mann-Whitney U) from the R programming language was used to determine if the mean of the average PIDs across the seven taxa of each milk and lactation gene set is statistically different from the whole genome. A two-sample Kolmogorov-Smirnov test was used to determine if the average PIDs of milk and mammary genes are drawn from the same distribution as the genome. Significance was determined by a *P*-value ≤ 0.05.

### Evolution analysis along the bovine lineage

For this analysis only, putative orthology was assigned using a three-way reciprocal best BLAST hit approach as implemented in the 'Ortholuge' program [58]. Ortholog sequences for the caseins and alpha-lactalbumin were manually curated. For each gene, d_N_/d_S _ratios were estimated from coding sequence alignments of the human-mouse-bovine orthologs by maximum likelihood using the codeml program from PAMLv4 [75]. Two models were implemented to test the statistical significance of variable selective pressures on each lineage. Under the one-ratio model, which acts as the null model (NSsites = 0, model = 0), each lineage was modeled to have the same d_N_/d_S _ratio. The ratio is constrained between 0 and 1, and does not allow for the presence of positive selection. The second model is a model of bovine-specific evolution, where the bovine lineage was selected as the 'foreground' lineage and d_N_/d_S _was specifically allowed to vary unconstrained on this lineage (model = 2). The two models were compared by likelihood ratio test, calculated from the log likelihood (lnL) values of both models. Twice the difference between lnL_model 2 _and lnL_one-ratio _was compared with a chi-square distribution to obtain the *P*-values.

## Abbreviations

d_N_: rate of non-synonymous substitutions per non-synonymous site; d_S_: rate of synonymous substitutions per synonymous site; EST: expressed sequence tag; LALBA: alpha-lactalbumin; LGB: beta-lactoglobulin; GO: Gene Ontology; PAEP: progestagen-associated endometrial protein; PID: percent identity; QTL: quantitative trait loci; UCSC: University of California at Santa Cruz.

## Authors' contributions

DGL and MR conceived of the study and participated in its design and coordination. DJL conducted evolutionary analyses. WFM and DGL performed genome localization analyses. EMZ and EVK provided orthology delineation. DGL, EVK, and NJM conducted phylogenetic experiments. JFM and GR curated milk protein orthologs and milk trait QTL. WCB produced the chromosome map. ASH created custom tracks for the UCSC Genome Browser. KS and RM conducted RT-PCR assays and prepared Additional data file 22. DGL produced the milk and mammary gene lists and conducted all other analyses. KSP supervised statistical analyses. DGL, TMC, MCN, AJM, RLT, and MR interpreted data. DGL, TMC, MCN, and MR drafted the manuscript. All authors contributed to and approved the final manuscript.

## Additional data files

The following additional data are available with the online version of this paper. Additional data file 1 is an Excel file with one spreadsheet listing the genes in each of the milk and mammary gene sets and their annotations. Additional data file 2 is a custom track in BED format for the UCSC Genome Browser, bovine assembly 3.1, that lists the genome locations of the milk protein gene set. Additional data file 3 is a custom track in BED format for the UCSC Genome Browser, bovine assembly 3.1, that lists the genome locations of the virgin mammary gene set. Additional data file 4 is a custom track in BED format for the UCSC Genome Browser, bovine assembly 3.1, that lists the genome locations of the pregnancy mammary gene set. Additional data file 5 is a custom track in BED format for the UCSC Genome Browser, bovine assembly 3.1, that lists the genome locations of the lactation mammary gene set. Additional data file 6 is a custom track in BED format for the UCSC Genome Browser, bovine assembly 3.1, that lists the genome locations of the involution mammary gene set. Additional data file 7 is a custom track in BED format for the UCSC Genome Browser, bovine assembly 3.1, that lists the genome locations of the mastitis mammary gene set. Additional data file 8 is an Excel file that lists genomic locations of curated milk trait QTL. Additional data file 9 is a custom track in BED format for the UCSC Genome Browser, bovine assembly 3.1, that lists genomic locations of curated milk trait QTL. Additional data file 10 is a Word document that provides additional analysis and discussion of milk trait QTL density. Additional data file 11 is an Excel file that lists candidate genes that occur within QTL with one spreadsheet per milk trait. Additional data file 12 is a custom track in BED format for the UCSC Genome Browser, bovine assembly 3.1, that lists genomic locations of candidate genes associated with the 'fat percentage' trait. Additional data file 13 is a custom track in BED format for the UCSC Genome Browser, bovine assembly 3.1, that lists genomic locations of candidate genes associated with the 'fat yield' trait. Additional data file 14 is a custom track in BED format for the UCSC Genome Browser, bovine assembly 3.1, that lists genomic locations of candidate genes associated with the 'milk yield' trait. Additional data file 15 is a custom track in BED format for the UCSC Genome Browser, bovine assembly 3.1, that lists genomic locations of candidate genes associated with the 'protein percentage' trait. Additional data file 16 is a custom track in BED format for the UCSC Genome Browser, bovine assembly 3.1, that lists genomic locations of candidate genes associated with the 'protein yield' trait. Additional data file 17 is an Excel file that lists the significant genomic clusters within each milk and mammary gene set. Additional data file 18 is a custom track in BED format for the UCSC Genome Browser, bovine assembly 3.1, that lists the genome locations of the significant genomic clusters. Additional data file 19 is an Excel file that lists accession numbers of mammalian orthologs of bovine milk protein genes. Additional data file 20 is a Word document that provides more detail on the conservation of milk protein genes in mammals. Additional data file 21 is an Excel spreadsheet that lists the EST libraries that were surveyed for this study, the number of ESTs in each library, a description of the physiological state of the animal and tissue used to derive the library, and, if the library was excluded from this study, the reason for exclusion. Additional data file 22 is a Word document that details the methods used to probe relative mRNA levels of *LGB-II*, *PCYOX1*, and *ART4 *in bovine mammary tissue at different stages of development and the RT-PCR results.

## Supplementary Material

Additional data file 1 Milk and mammary gene sets. Click here for file

Additional data file 2 Custom track in BED format for the UCSC Genome Browser, bovine assembly 3.1. Click here for file

Additional data file 3 Custom track in BED format for the UCSC Genome Browser, bovine assembly 3.1. Click here for file

Additional data file 4 Custom track in BED format for the UCSC Genome Browser, bovine assembly 3.1. Click here for file

Additional data file 5 Custom track in BED format for the UCSC Genome Browser, bovine assembly 3.1. Click here for file

Additional data file 6 Custom track in BED format for the UCSC Genome Browser, bovine assembly 3.1. Click here for file

Additional data file 7 Custom track in BED format for the UCSC Genome Browser, bovine assembly 3.1. Click here for file

Additional data file 8 Genomic locations of curated milk trait QTL Click here for file

Additional data file 9 Custom track in BED format for the UCSC Genome Browser, bovine assembly 3.1. Click here for file

Additional data file 10 Additional analysis and discussion of milk trait QTL density Click here for file

Additional data file 11 Candidate genes that occur within QTL with one spreadsheet per milk trait Click here for file

Additional data file 12 Custom track in BED format for the UCSC Genome Browser, bovine assembly 3.1. Click here for file

Additional data file 13 Custom track in BED format for the UCSC Genome Browser, bovine assembly 3.1. Click here for file

Additional data file 14 Custom track in BED format for the UCSC Genome Browser, bovine assembly 3.1. Click here for file

Additional data file 15 Custom track in BED format for the UCSC Genome Browser, bovine assembly 3.1. Click here for file

Additional data file 16 Custom track in BED format for the UCSC Genome Browser, bovine assembly 3.1. Click here for file

Additional data file 17 Significant genomic clusters within each milk and mammary gene set Click here for file

Additional data file 18 Custom track in BED format for the UCSC Genome Browser, bovine assembly 3.1. Click here for file

Additional data file 19 Accession numbers of mammalian orthologs of bovine milk protein genes Click here for file

Additional data file 20 More detail on the conservation of milk protein genes in mammals Click here for file

Additional data file 21 EST libraries that were surveyed for this study, the number of ESTs in each library, a description of the physiological state of the animal and tissue used to derive the library, and, if the library was excluded from this study, the reason for exclusion. Click here for file

Additional data file 22 Methods used to probe relative mRNA levels of *LGB-II*, *PCYOX1*, and *ART4 *in bovine mammary tissue at different stages of development and the RT-PCR results. Click here for file
