# Supplementary material for: The bovine lactation genome: insights into the evolution of mammalian milk
Source: Genome Biol. 2009 Apr 24;10(4):R43. doi: 10.1186/gb-2009-10-4-r43 (PMC2688934; doi:10.1186/gb-2009-10-4-r43)
Supplement: Additional data file 10 — Additional analysis and discussion of milk trait QTL density [file gb-2009-10-4-r43-S10.doc]

**Milk trait QTL Density Details**

While all 29 autosomes have at least one milk trait QTL, the density of these QTL is variable across the chromosomes (Suppl. Figure 1). The chromosomes with the highest density of QTLs are chromosomes 27, 6, 20, and 14. Separated by trait in Suppl. Figures 2-6, it is apparent that all five of these traits are affected by multiple genes. Possible differences in genetic architecture are most obvious between fat and protein percentage traits where fat percentage may be controlled by relatively fewer genes of larger effects while protein percentage may be controlled by far more genes with smaller effects.

Supplemental Figure 1: Milk trait QTL density

Supplemental Figure 2: Milk Yield QTL density

Supplemental Figure 3: Milk Fat Yield QTL density

Supplemental Figure 4: Milk Protein Yield QTL density

Supplemental Figure 5: Milk Fat Percentage QTL density

Supplemental Figure 6: Milk Protein Percentage QTL density
