# Supplementary material for: The bovine lactation genome: insights into the evolution of mammalian milk
Source: Genome Biol. 2009 Apr 24;10(4):R43. doi: 10.1186/gb-2009-10-4-r43 (PMC2688934; doi:10.1186/gb-2009-10-4-r43)
Supplement: Additional data file 18 — Custom track in BED format for the UCSC Genome Browser, bovine assembly 3.1. [file gb-2009-10-4-r43-S18.bed]

```
track name=cluster_loc_062708 description='Statistically significant clusters (June 27)' db=bosTau3 color=255,155,0
chr10	39954093	40454093
chr10	31360707	31860707
chr10	31641344	32141344
chr10	31460201	31960201
chr10	79479178	79979178
chr10	91356062	91856062
chr10	64099729	64599729
chr10	80008355	80508355
chr11	63305058	63805058
chr11	21351267	21851267
chr11	93289904	93789904
chr11	93335500	93835500
chr11	93451861	93951861
chr11	93289904	93789904
chr11	45294573	45794573
chr11	94387439	94887439
chr11	36865489	37365489
chr11	100495406	100995406
chr11	6174162	6674162
chr11	11454703	11954703
chr11	100442538	100942538
chr11	94806796	95306796
chr11	94487970	94987970
chr11	63440043	63940043
chr11	93924771	94424771
chr11	94092250	94592250
chr11	94124469	94624469
chr11	94154826	94654826
chr11	63305058	63805058
chr11	94387439	94887439
chr11	94806796	95306796
chr12	14325676	14825676
chr12	69035431	69535431
chr12	28494895	28994895
chr12	16975133	17475133
chr13	27103211	27603211
chr13	56605219	57105219
chr13	74230602	74730602
chr13	62021873	62521873
chr13	27173233	27673233
chr13	74331353	74831353
chr13	65541740	66041740
chr13	44132975	44632975
chr13	39251035	39751035
chr13	27103211	27603211
chr13	27173233	27673233
chr13	66267625	66767625
chr13	76561237	77061237
chr13	76652341	77152341
chr13	73755256	74255256
chr14	92481	592481
chr14	266582	766582
chr14	499535	999535
chr14	80204	580204
chr14	396497	896497
chr14	478848	978848
chr14	499535	999535
chr14	259683	759683
chr14	489553	989553
chr14	22893061	23393061
chr14	799829	1299829
chr14	35160730	35660730
chr14	18226229	18726229
chr14	64752593	65252593
chr14	92481	592481
chr14	396497	896497
chr14	64968516	65468516
chr14	17914258	18414258
chr15	44498653	44998653
chr15	37848519	38348519
chr15	56943824	57443824
chr15	37660428	38160428
chr15	34020598	34520598
chr15	34254792	34754792
chr15	56582906	57082906
chr15	56264942	56764942
chr15	37660428	38160428
chr15	22024913	22524913
chr15	31505242	32005242
chr15	40875383	41375383
chr15	35801176	36301176
chr15	56582906	57082906
chr15	56898328	57398328
chr15	56264942	56764942
chr15	57130381	57630381
chr15	57350892	57850892
chr15	37848519	38348519
chr15	58970401	59470401
chr16	26483721	26983721
chr16	20447323	20947323
chr16	61143158	61643158
chr16	22977940	23477940
chr16	23063993	23563993
chr16	52252680	52752680
chr17	68409803	68909803
chr17	68623030	69123030
chr17	67419886	67919886
chr17	68409803	68909803
chr17	2253354	2753354
chr17	67419886	67919886
chr17	4136302	4636302
chr17	68409803	68909803
chr17	47249091	47749091
chr17	68562128	69062128
chr17	49054478	49554478
chr17	3809506	4309506
chr17	68623030	69123030
chr17	49656693	50156693
chr18	35807777	36307777
chr18	49432757	49932757
chr18	23685458	24185458
chr18	32230509	32730509
chr18	32869740	33369740
chr18	32702716	33202716
chr18	47858860	48358860
chr18	7328874	7828874
chr19	50430377	50930377
chr19	19571631	20071631
chr19	37988467	38488467
chr19	44544435	45044435
chr19	38019071	38519071
chr19	49980031	50480031
chr19	50171832	50671832
chr19	41429367	41929367
chr19	41474370	41974370
chr19	61375903	61875903
chr19	50073716	50573716
chr19	41166611	41666611
chr19	41012094	41512094
chr19	50430377	50930377
chr19	52606719	53106719
chr19	52648848	53148848
chr19	25524712	26024712
chr19	40413973	40913973
chr19	40672828	41172828
chr19	52606719	53106719
chr1	76731930	77231930
chr1	134070747	134570747
chr1	131075600	131575600
chr1	97915495	98415495
chr1	97915495	98415495
chr1	133496888	133996888
chr1	76724285	77224285
chr1	64018013	64518013
chr1	131763258	132263258
chr1	64018013	64518013
chr1	134070747	134570747
chr1	59376187	59876187
chr20	35432146	35932146
chr21	21385466	21885466
chr21	25940160	26440160
chr21	62574665	63074665
chr22	57913090	58413090
chr22	48745536	49245536
chr22	48734987	49234987
chr22	54544519	55044519
chr22	49162738	49662738
chr22	48745536	49245536
chr22	57664604	58164604
chr22	48504109	49004109
chr22	48745536	49245536
chr22	48009196	48509196
chr22	48535205	49035205
chr22	9582264	10082264
chr22	57664604	58164604
chr22	52191735	52691735
chr22	48901195	49401195
chr22	57626677	58126677
chr22	48535205	49035205
chr22	9582264	10082264
chr22	52191735	52691735
chr22	48910855	49410855
chr23	7181416	7681416
chr23	20848206	21348206
chr23	47057965	47557965
chr23	5275982	5775982
chr23	22214892	22714892
chr23	20848206	21348206
chr23	22032975	22532975
chr23	21229634	21729634
chr23	20848206	21348206
chr23	21229634	21729634
chr23	14569791	15069791
chr23	47057965	47557965
chr23	5594858	6094858
chr23	14458647	14958647
chr23	5022681	5522681
chr23	21229634	21729634
chr23	20111446	20611446
chr23	24111104	24611104
chr23	24058989	24558989
chr23	20848206	21348206
chr23	5093655	5593655
chr23	5098405	5598405
chr23	5275982	5775982
chr23	24203732	24703732
chr23	20803359	21303359
chr23	24219386	24719386
chr23	14770892	15270892
chr23	24280456	24780456
chr24	35399977	35899977
chr24	41212832	41712832
chr24	39587990	40087990
chr24	41289631	41789631
chr24	41368916	41868916
chr24	39657903	40157903
chr24	39903518	40403518
chr24	41212832	41712832
chr24	41368916	41868916
chr24	41289631	41789631
chr24	20093586	20593586
chr24	48233440	48733440
chr24	45856756	46356756
chr24	48312570	48812570
chr25	195197	695197
chr25	32974153	33474153
chr25	285628	785628
chr25	285628	785628
chr25	24734003	25234003
chr25	38146052	38646052
chr25	3922995	4422995
chr25	3922995	4422995
chr26	30161676	30661676
chr26	30161676	30661676
chr26	15997830	16497830
chr26	11333821	11833821
chr27	23649117	24149117
chr27	17273479	17773479
chr27	32571140	33071140
chr27	32269401	32769401
chr27	32229235	32729235
chr28	23698781	24198781
chr28	12213576	12713576
chr28	6314368	6814368
chr28	25472920	25972920
chr28	24397551	24897551
chr28	28278897	28778897
chr28	24397551	24897551
chr28	6359209	6859209
chr28	11655347	12155347
chr29	32988873	33488873
chr29	32988873	33488873
chr29	39818103	40318103
chr29	32689659	33189659
chr29	32689659	33189659
chr29	37701088	38201088
chr29	36231329	36731329
chr29	32970552	33470552
chr29	36411074	36911074
chr29	23457418	23957418
chr2	66771861	67271861
chr2	116982934	117482934
chr2	16824153	17324153
chr2	110880079	111380079
chr2	16824153	17324153
chr2	116720087	117220087
chr2	17060763	17560763
chr2	110880079	111380079
chr2	79062403	79562403
chr2	82583415	83083415
chr2	95237706	95737706
chr2	115444210	115944210
chr2	115491747	115991747
chr3	13257588	13757588
chr3	101832551	102332551
chr3	102150714	102650714
chr3	101594684	102094684
chr3	13223504	13723504
chr3	13078603	13578603
chr3	98048066	98548066
chr3	31893339	32393339
chr3	14080965	14580965
chr3	91878930	92378930
chr3	17060597	17560597
chr3	17279928	17779928
chr3	16224625	16724625
chr3	17165734	17665734
chr3	14155284	14655284
chr3	14505346	15005346
chr3	12665520	13165520
chr3	15468594	15968594
chr3	17492684	17992684
chr3	17608705	18108705
chr3	17445274	17945274
chr3	17730276	18230276
chr3	17701053	18201053
chr3	16672945	17172945
chr3	12013562	12513562
chr3	91878930	92378930
chr3	106985971	107485971
chr3	16742701	17242701
chr3	786302	1286302
chr3	77708536	78208536
chr3	52181705	52681705
chr3	106985971	107485971
chr3	17892762	18392762
chr3	19737370	20237370
chr3	12127417	12627417
chr4	68406004	68906004
chr4	26236309	26736309
chr4	90817026	91317026
chr4	105205352	105705352
chr4	68168670	68668670
chr4	26236309	26736309
chr4	104859507	105359507
chr4	68406004	68906004
chr4	62784157	63284157
chr4	68406004	68906004
chr4	91406928	91906928
chr4	68168670	68668670
chr4	68536742	69036742
chr4	86508443	87008443
chr4	90951360	91451360
chr4	62784157	63284157
chr4	91406928	91906928
chr4	63118414	63618414
chr4	98827187	99327187
chr5	73674907	74174907
chr5	107830336	108330336
chr5	108305223	108805223
chr5	114878177	115378177
chr5	56160154	56660154
chr5	10199221	10699221
chr5	105612873	106112873
chr5	30945952	31445952
chr5	55853205	56353205
chr5	27475834	27975834
chr5	55900851	56400851
chr6	33327235	33827235
chr6	53965394	54465394
chr6	89544715	90044715
chr6	33327235	33827235
chr6	53965394	54465394
chr6	79528410	80028410
chr6	79291030	79791030
chr6	79528410	80028410
chr6	54112500	54612500
chr6	89544715	90044715
chr6	105538557	106038557
chr7	16436762	16936762
chr7	57516680	58016680
chr7	14573783	15073783
chr7	34985539	35485539
chr7	10451522	10951522
chr7	16429645	16929645
chr7	35290708	35790708
chr7	35241376	35741376
chr7	35226278	35726278
chr7	42387001	42887001
chr7	351710	851710
chr7	57516680	58016680
chr7	34985539	35485539
chr7	9757814	10257814
chr7	42186654	42686654
chr7	1559373	2059373
chr7	9757814	10257814
chr7	42428955	42928955
chr7	36084339	36584339
chr7	351710	851710
chr7	50363286	50863286
chr7	35295033	35795033
chr7	42343228	42843228
chr7	41999352	42499352
chr7	14565072	15065072
chr7	2890573	3390573
chr7	57360454	57860454
chr7	35733588	36233588
chr7	42428955	42928955
chr7	12066932	12566932
chr7	50148926	50648926
chr8	58701817	59201817
chr8	72280213	72780213
chr8	9284567	9784567
chr8	71995538	72495538
chr8	77647317	78147317
chr8	77647317	78147317
chrX	91097953	91597953
chrX	91097953	91597953
chrX	59377654	59877654
chrX	32081873	32581873
chrX	32981140	33481140
chrX	31923262	32423262
chrX	9045734	9545734
chrX	62013922	62513922
chrX	99278272	99778272
chrX	12876849	13376849
chrX	32081873	32581873
chrX	32981140	33481140
chrX	9057229	9557229
```
