# Supplementary material for: The bovine lactation genome: insights into the evolution of mammalian milk
Source: Genome Biol. 2009 Apr 24;10(4):R43. doi: 10.1186/gb-2009-10-4-r43 (PMC2688934; doi:10.1186/gb-2009-10-4-r43)
Supplement: Additional data file 20 — More detail on the conservation of milk protein genes in mammals [file gb-2009-10-4-r43-S20.doc]

### Conservation of milk proteins genes in mammals

For appropriate comparisons to the whole genome which is necessarily uncurated, the uncurated orthologs of the Milk Protein Gene Set are used, only one-third of which comprises single copy orthologs across all seven mammalian taxa. To increase the sample size of milk protein genes used to examine conservation in mammals, the pairwise PID between human and each of cow, mouse, opossum, and platypus was individually assessed, requiring, in each case, that orthologs be single copies only in bovine and the two taxa being compared. Here, milk protein sequences are statistically more conserved between human and other mammals than the products of other genes in the genome. The pairwise PID distributions between human and other mammals suggest a subset of milk proteins are distinctly more conserved than genome-wide (see Supplementary Figures 7-10 below). The human-cow distribution is most dramatically different from the whole genome, as a full quarter of the set of 137 milk protein genes with single copies in these two genomes are very highly conserved with a pairwise PID of 97.5?% or greater.

**Supplemental Figure 7: Milk protein conservation between human and platypus**

**Supplemental Figure 8: Milk protein conservation between human and opossum**

**Supplemental Figure 9: Milk protein conservation between human and mouse**

**Supplemental Figure 10: Milk protein conservation between human and cow**
