# Supplementary material for: The bovine lactation genome: insights into the evolution of mammalian milk
Source: Genome Biol. 2009 Apr 24;10(4):R43. doi: 10.1186/gb-2009-10-4-r43 (PMC2688934; doi:10.1186/gb-2009-10-4-r43)
Supplement: Additional data file 22 — Methods used to probe relative mRNA levels of LGB-II, PCYOX1, and ART4 in bovine mammary tissue at different stages of development and the RT-PCR results. [file gb-2009-10-4-r43-S22.doc]

**RT-PCR Methods and Results**

**MATERIALS AND METHODS**

***Animals***

The lactating (6 hours post milking) and 8 day involution samples are a subset of this trial that we have described previously (Singh et al., 2005; 2008) :

Involution of the bovine mammary gland was induced by abrupt termination of milking in 48 non-pregnant Friesian dairy cows at, or close to, their peak milk production and before mating in mid-lactation (average DIM, 92 ± 3). The primiparous cows were solely pasture-fed, were milked twice daily from parturition and had an average daily milk yield of 14.3 ± 0.3 kg/cow. The average SCC in composite (four quarters) milk prior to the termination of milking was 159,000 ± 20,000 cells/ml. The animals were slaughtered at the Ruakura abattoir (Hamilton, NZ) using standard commercial procedures (electrical stunning followed by exsanguination) at 0, 6, 12, 18, 24, 36, 72 and 192 h (n = 6 per group) after the last milking. Mammary alveolar tissue (approximately 30 g) was collected from the middle of the upper one-third of the gland of a rear quarter of each animal and snap-frozen in liquid nitrogen for subsequent RNA and protein extraction. Animal experimentation was conducted in compliance with the rules and guidelines of the Ruakura Animal Ethics committee.

The following samples have only brief descriptions: Virgin - 2-2.5 year old beef cows, Angus cross and Friesian/Hereford; 30 day prepartum - beef cows, pregnant, 30 days prepartum, shorthorn cross; 85 day prepartum - beef cows, pregnant 85 days prepartum / 195 day pregnant, Angus; 20 day involuting - beef cow, Angus-cross, 5 years old; and a dried off cow.

***Quantitative real-time reverse transcription-polymerase chain reaction analysis (RT-PCR)***

Total RNA was extracted from ground tissue using TRIzol (Invitrogen, Carlsbad, California, USA) and 1 μg was treated with 1 U DNase I (Invitrogen). Samples were further purified through RNeasy gel columns (Qiagen Sciences, Maryland, USA) and converted to cDNA using a SuperScript II First-Strand Synthesis system as described by the manufacturer (Invitrogen). The cDNA products were diluted 10-fold and samples (1 µl) were assayed in duplicate, by real-time quantitative RT-PCR using an ABI PRISM 7900 Sequence Detection System (PE Applied Biosystems, Foster City, California, USA). Detection of the product was by SYBR Green I (Morrison et al., 1998), using the Universal PCR Master Mix (PE Applied Biosystems). For each assay two control reactions were included; a reverse transcriptase-negative control, and omission of the template (no template control). Any amplification occurring in these control reactions would indicate the presence of non-RNA template. PCR primer sequences for detection of LGB-II, PCYOX1 and ART4 were designed using Primer3 (Rozen and Skaletsky, 1998). Primer sequences and conditions for amplification are listed in Table 1. The thermal cycling programs were 95 °C for 10 min followed by 40 cycles of 95 °C for 15 sec, 60 °C for 30 sec and 72 °C for 30 sec. Ubiquitin was used as an internal control. Dissociation curve analysis confirmed a single product. The threshold cycle (CT) number for each gene generated by real-time RT-PCR was used to quantify the relative abundance of each gene using the relative standard curve method (PE Applied Biosystems, Sequence Detection System, Chemistry Guide, 2003). The values for each gene were log10-transformed and normalized to ubiquitin log10-transformed values.

Products have not yet been verified by sequencing.

***Statistical Analysis***

The differences between means were analyzed using ANOVA in the Minitab software package (Minitab Inc., 2003). The means for each group were backtransformed and expressed as the fold change ± SEM relative to the virgin mean.

**RESULTS**

The PCYOX1 and ART4 mRNA levels were not differentially expressed in alveolar mammary tissue from virgin, prepartum, lactating, involuting and dried off cows (Figure 1 and 2, respectively). LGB-II could not be detected in these samples. Two sets of primers were test for LGB-II and neither set worked.

Figure 1

Figure 2

**Table 1.** Primer sequences, primer concentrations, product sizes and annealing temperatures used for measurement of gene expression in alveolar tissue of the bovine mammary gland by quantitative real-time RT-PCR analysis.

| Gene name^1^ | Forward Primer (F)  Reverse Primer (R)  5’ 🡪 3’ | Product size, bp | Annealing temp, °C | Primer concentration nM |
| --- | --- | --- | --- | --- |
| SYBER Green PCR | | | | |
| PCYOX1 | F: gcacttcagcagcctattatctg  R: tcatgagactggtagcggtagat | 382 | 60 | 100 |
| ART4 | F: ctgcttcaaggggattatttcat  R: ctcgagacaatctctttcctcag | 302 | 60 | 100 |
| LGB-II | F: agtgtgtggagaagaccctcat  R: ggtggtcacagaaaggtgct | 365 | 60 | 100 |
| LGB-II | F: cagactacagcagctacacgatct  R: gccttcctcctcttttcctg | 295 | 60 | 100 |

^1^mono (ADP-ribosyl)transferase (ART4), prenylcysteine oxidase 1 (PCYOX1), *β*-lactoglobulin-II (LGB-II)

***References***

Singh, K., Davis, S.R., Dobson, J., Molenaar, A.J., Wheeler, T., Prosser, C., Farr, V.C., Oden, K., Swanson, K., Henderson, H., Wilson, T., Stelwagen, K. 2008. cDNA microarray analysis reveals antioxidant and immune genes are up-regulated during involution of the bovine mammary gland. Journal of Dairy Science. 91(6):2236-46.

Singh K., Dobson J, Cooper C, Davis S, Farr V, Molenaar A, Stelwagen K 2005 Milk accumulation decreases expression of genes involved in ECM communication and initiates apoptosis in the bovine mammary gland. *Livest. Prod. Sci.* 98: 67-78

Steve Rozen, Helen J. Skaletsky (1998) Primer3. Code available at <http://www-genome.wi.mit.edu/genome_software/other/primer3.html>.
